# Facial Skin With Conspicuous Enlarged Pores Closely Related to Severity of Facial Acneiform Rash and Therapeutic Effects of EGFR Inhibitors in RAS Wild‐Type Metastatic Colorectal Cancer: Ancillary Analysis of FAEISS Study (NCCH1512)

**DOI:** 10.1111/1346-8138.17813

**Published:** 2025-06-12

**Authors:** Shusuke Yoshikawa, Katsuko Kikuchi, Keiko Nozawa, Yasuko Nakai, Haruhiko Fukuda, Taro Shibata, Ryunosuke Machida, Tetsuya Hamaguchi, Atsuo Takashima, Sumiko Takatsuka, Tomohiro Nishina, Akihito Kawazoe, Takahiro Tsushima, Masanobu Takahashi, Akiko Hasegawa, Toshiki Masuishi, Naoya Yamazaki, Yoshio Kiyohara

**Affiliations:** ^1^ Dermatology Division Shizuoka Cancer Center Hospital Shizuoka Japan; ^2^ Sendai Taihaku Dermatology Clinic Sendai Japan; ^3^ Department of Nursing, Faculty of Nursing Mejiro University Faculty of Nursing Iwatsuki Japan; ^4^ Nakai Dermatology Clinic Tsu Japan; ^5^ Clinical Research Support Office, National Cancer Center Hospital Tokyo Japan; ^6^ Department of Medical Oncology Saitama Medical University International Medical Center Saitama Japan; ^7^ Department of Gastrointestinal Medical Oncology National Cancer Center Hospital Tokyo Japan; ^8^ Department of Dermatology Niigata Cancer Center Hospital Niigata Japan; ^9^ Department of Gastrointestinal Medical Oncology National Hospital Organization Shikoku Cancer Center Matsuyama Japan; ^10^ Department of Gastrointestinal Oncology National Cancer Center Hospital East Kashiwa Japan; ^11^ Division of Gastrointestinal Oncology Shizuoka Cancer Center Hospital Shizuoka Japan; ^12^ Department of Medical Oncology Tohoku University Hospital Sendai Japan; ^13^ Department of Medical Oncolog Osaka International Cancer Institute Osaka Japan; ^14^ Department of Clinical Oncology Aichi Cancer Center Hospital Nagoya Japan; ^15^ Department of Dermatologic Oncology National Cancer Center Hospital Tokyo Japan

**Keywords:** acneiform rash, colorectal cancer, enlarged pores, epidermal growth factor receptor (EGFR), facial skin type

## Abstract

Prophylactic treatment with oral minocycline or doxycycline, moisturizers, and sunscreens has been reported to be beneficial for acneiform rash (AfR) caused by epidermal growth factor receptor (EGFR) inhibitors. It is desirable to predict which patients may develop severe AfR and provide prophylactic treatment. This study aimed to evaluate the association between the worst grade of facial AfR (FAfR) after initiating therapy with EGFR inhibitors and the characteristic skin type in patients with RAS wild‐type metastatic colorectal cancer enrolled in the FAEISS study (a phase III, open‐label, randomized trial evaluating topical corticosteroids for Facial Acneiform dermatitis by EGFR Inhibitors: Stepwise rank down from potent corticosteroids). Using pretreatment photographs of the face, characteristic skin types, including enlarged pores, oiliness (greasiness), and skin color/redness, were graded on a scale of 1–3. Grade 2 or higher FAfR developed in 9.1%, 27.0%, and 45.8% of patients with enlarged pore scores of 1, 2, and 3, respectively. Patients with enlarged pores tended to have more severe FAfR (*p* = 0.0216). Moreover, the disease control rate (complete remission/partial remission/stable disease) of the primary disease was seen in 59.1%, 70.6%, and 87.0% of patients with an enlarged pore score of 1, 2, and 3, respectively, showing a statistically significant trend (*p* = 0.038). This study suggests that a facial skin type with a high number of enlarged pores may be a marker for predicting AfR risk due to anti‐EGFR antibody therapy and better therapeutic effects for RAS wild‐type metastatic colorectal cancer.

**Abbreviations**
AfR: acneiform rash
CR: complete remission
EGFR: epidermal growth factor receptor
FAfR: facial acneiform rash
PR: partial remission
SD: stable disease

## 1 Introduction

Acneiform rash (AfR) is an adverse event that occurs in response to a high number of epidermal growth factor receptor (EGFR) inhibitors. Survival rates have been reported to be higher in colorectal cancer patients treated with cetuximab who develop more severe AfR [1], suggesting that the severity of AfR may be indicative of the EGFR inhibitor's efficacy against cancer. However, AfR, particularly facial acneiform rash (FAfR), is a bothersome adverse event that reduces patients' quality of life. Prophylactic treatment of AfR with oral doxycycline or minocycline, weak topical steroids, moisturizers, and sunscreen has been reported to be beneficial for patients receiving panitumumab, an anti‐EGFR antibody drug [2, 3]. Additionally, Nishino et al. reported that prophylactic treatment with oral minocycline and moisturizers was useful in preventing the onset of disease in patients with non‐small cell lung cancer receiving EGFR/TKIs [4].

From the perspective of managing adverse events in patients undergoing cancer treatment, prophylactic therapy should be applied to high‐risk patients with FAfR rather than to all patients treated with EGFR inhibitors. Kikuchi et al. reported that younger (≤ 60 years) age and higher skin erythema index and skin surface lipid level on the face, as measured noninvasively before treatment with EGFR inhibitors, tend to exacerbate FAfR [5]. Recently, we reported the results of the FAEISS study (a phase III, open‐label, randomized trial evaluating topical corticosteroids for Facial Acneiform dermatitis by EGFR Inhibitors: Stepwise rank down from potent corticosteroids; NCCH1512, registered in the UMIN Clinical Trials Registry [www.umin.ac.jp/ctr/] as UMIN000024113), in which FAfR was treated with oral minocycline and a topical moisturizer followed by topical steroid therapy in patients with colorectal cancer [6]. The current study is an ancillary analysis of the FAEISS study, which was designed to identify high‐risk patients prior to therapy with EGFR inhibitors by examining the association between pretreatment facial skin type (i.e., enlarged pores, oiliness, and skin color/redness) and the subsequent FAfR grade.

## 2 Methods

### 2.1 Patients

Eligible patients were those with *RAS* wild‐type metastatic CRC that was determined to be unresectable or had relapsed after curative resection. The patients were treated with cetuximab or panitumumab alone or in combination with cytotoxic agents. Other eligibility criteria included age 20–79 years; an Eastern Cooperative Oncology Group Performance Status [7] score of 0 or 1; absence of active bacterial, fungal, and viral infections; and absence of facial lesions influencing the evaluation of facial lesions. Informed consent for participation in this prospective study was obtained before enrollment. Patients with a history of anti‐EGFR therapy were excluded.

### 2.2 Study Design

This was an ancillary analysis of the FAEISS study, an open‐label, multicenter, randomized controlled trial [6]. Eligible patients started preemptive therapy with oral minocycline at 100 or 200 mg daily and applied a skin moisturizer containing heparinoid to the face twice daily from the start of anti‐EGFR antibody therapy. All patients were instructed to avoid solar ultraviolet radiation, and the use of sunscreen was recommended. Patients who developed grade 1 or 2 FAfR within 8 weeks of initiating anti‐EGFR antibody therapy received an eight‐week course of topical corticosteroid treatment. Steroid therapy was administered according to two protocols: a ranking‐up group, in which corticosteroid potency was escalated every 2 weeks starting from a weak topical corticosteroid, and a ranking‐down group, in which potency was de‐escalated every 2 weeks starting from a very strong topical corticosteroid. The attending physician graded FAfR at weeks 0, 2, 4, 6, and 8 of corticosteroid therapy according to the qualitative scheme described by Scope et al. [8], with minor modifications. FAfR was classified as follows: grade 0, no symptoms; grade 1, less than one‐third of the face involved; grade 2, more than one‐third but less than two‐thirds of the face involved; and grade 3, more than two‐thirds of the face involved. For analysis, the highest FAfR grade observed in each patient during the corticosteroid treatment period was used. Topical corticosteroids of strong or very strong classes were used for AfR in non‐facial areas. If grade 3 or higher AfR affecting the whole body, according to the Common Terminology Criteria for Adverse Events version 4.0‐Japan Clinical Oncology Group, was observed, the protocol treatment was terminated.

### 2.3 Evaluations

**FIGURE 1:**
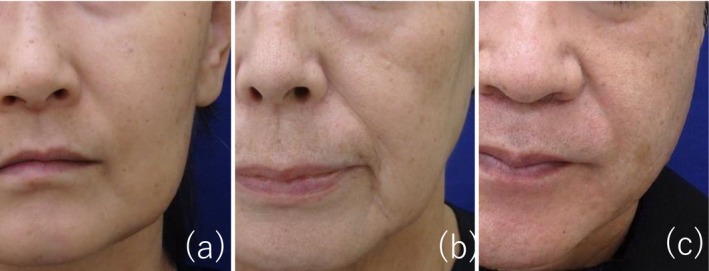
Reference photos of score 1 (a), score 2 (b), and score 3 (c) of enlarged pores.

Digital photographs of the face were taken using a predetermined standardized method on the respective evaluation dates before and after treatment. Using pretreatment photographs of the face, characteristic skin types, including enlarged pores, oiliness (greasiness), and skin color/redness, were graded using a score of 1–3 by three board‐certified dermatologists (central review). Enlarged pores were assessed as follows: score 1, no enlarged pores with a smooth skin surface; score 2, enlarged pores visible on the nose and inner part of the cheeks; and score 3, enlarged pores visible on the nose and lateral part of the cheeks with thick skin (Figure 1). Oiliness (greasiness) was scored as follows: 1, slight; 2, moderate; and 3, intense (severe). Skin color/redness was scored as follows: 1, slight erythema; score 2, moderate erythema; and 3, intense (severe) erythema.

### 2.4 Statistical Analyses

The proportion of grade 2 or higher FAfR, disease control rate (DCR), and their 95% confidence interval (CIs) were calculated for each skin type score. The CIs were estimated using the Clopper‐Pearson method. The proportions of grade 2 or higher FAfR between the scores for each skin type were compared using Fisher's exact test. The tendency of the DCR based on the scores for each skin type was evaluated using the Cochran‐Armitage trend test. The DCRs between grade 2 or higher and grade 1 or lower FAfR were compared using Fisher's exact test. All P‐values were two‐sided. Statistical analyses were performed using SAS version 9.4 software (SAS Institute, Cary, NC).

## 3 Results

### 3.1 Patients

Between October 2016 and December 2018, 172 patients with RAS wild‐type metastatic colorectal cancer were enrolled and received preemptive therapy. Excluding non‐evaluable patients and those with deficiencies, the age‐sex profiles of the patients (*n* = 150) by facial skin type are shown in Table 1. The scores for enlarged pores, oiliness, and skin color (redness) did not change with age. The scores for enlarged pores tended to be higher in males than in females. Among them, we analyzed 146 patients for associations between facial skin types and FAfR and 147 patients for the best overall response, omitting patients with unevaluable data.

**TABLE 1:** Patients profile by facial skin type; enlarged pores, oiliness and redness.

	Enlarged pores			Oiliness (greasiness)			Skin color/redness		
	Score 1	Score 2	Score 3	Score 1	Score 2	Score 3	Score 1	Score 2	Score 3
	(*n* = 22)	(*n* = 104)	(*n* = 24)	(*n* = 0)	(*n* = 116)	(*n* = 34)	(*n* = 0)	(*n* = 113)	(*n* = 37)
Age (year)									
Median (range)	58 (26–76)	65 (30–79)	58 (38–73)	—	62 (26–79)	63 (38–76)	—	62 (26–79)	63 (38–78)
Sex no. (%)									
Male	6 (6.6)	64 (70.3)	21 (23.1)	—	58 (100)	0 (0.0)	—	56 (61.5)	35 (38.5)
Female	16 (27.1)	40 (67.8)	3 (5.1)	—	57 (62.6)	34 (37.4)	—	57 (96.6)	2 (3.4)

### 3.2 Association Between Facial Skin Type and FAfR

Grade 2 or higher FAfR developed in 9.1%, 27.0%, and 45.8% of patients with enlarged pore score 1, 2, and 3, respectively. Patients with enlarged pores tended to have higher FAfR (*p* = 0.0216, Fisher's exact test) (Table 2). There was no consistent trend between the degree of oiliness or redness score and the FAfR grade.

**TABLE 2:** Association between skin type and the worst grade of FAfR.

Enlarged pores	No. of grade 2 or higher facial AfR	% grade 2 or higher facial AfR (95% CI)	*p*
Score 1			
Total	2/22	9.1 (1.1‐29.2)	—
Male	1/6	16.7 (0.4‐64.1)	
Female	1/16	6.3 (0.2‐30.2)	
Score 2			
Total	27/100	27.0 (18.6‐36.8)	—
Male	19/61	31.1 (19.9‐44.3)	
Female	8/39	20.5 (9.3‐36.5)	
Score 3			
Total	11/24	45.8 (25.6‐67.2)	0.0216^a^
Male	10/21	47.6 (25.7‐70.2)	0.3038
Female	1/3	33.3 (0.8‐90.6)	0.2433

Abbreviations: AfR, acneiform rash; CI, confidence interval.

^a^Shows the relationship between pre‐existing enlarged pores and the frequency of acneiform rash.

### 3.3 Association Between Facial Skin Type and Best Overall Response to EGFR Inhibitors

The response of the primary disease to EGFR inhibitor therapy, assessed as % PR or SD, was 59.1%, 70.6%, and 87.0% in patients with enlarged pore scores 1, 2, and 3, respectively. A higher score for enlarged pores was significantly associated with a better response to EGFR inhibitors (*p* = 0.038, Cochran‐Armitage trend test) (Table 3). No significant differences were found between the redness or oiliness scores and the grade of FAfR (data not shown).

**TABLE 3:** Association between skin type (enlarged pores) and best overall response.

Enlarged pores	No. of PR or SD/*N*	% PR or SD	*p*
		(95% CI)	
Score 1	13/22	59.1	—
		(36.4–79.3)	
Score 2	72/102	70.6	—
		(60.8–79.2)	
Score 3	20/23	87.0	0.038
		(66.4–97.2)	

Abbreviations: CI, confidence interval; PR, partial remission; SD, stable disease.

### 3.4 Association Between the Worst Grade of FAfR and Best Overall Response to EGFR Inhibitors

The response of the primary disease to EGFR inhibitor therapy, assessed as % PR or SD, was 84.1% in patients with a worst FAfR grade of 2 or higher and 73.6% in patients with a worst FAfR grade of less than 2. No significant trend was observed (*p* = 0.2139, Fisher's exact test) (Table 4).

**TABLE 4:** Association between the worst grade of FAfR and best overall response.

	No. of PR or SD/*N*	%PR or SD	*p*
		(95% CI)	
< grade 2	89/121	73.6	—
		(64.8–81.2)	
> = grade 2	37/44	84.1	0.2139
		(69.9–93.4)	

Abbreviations: CI, confidence interval; FAfR, facial acneiform rash; PR, partial remission; SD, stable disease.

## 4 Discussion

AfR is a well‐known skin toxicity caused by EGFR inhibitors, which primarily develops in seborrheic areas such as the scalp, face, neck, upper chest, and upper back. Inhibition of EGFR signaling in basal keratinocytes of the epidermis and pilosebaceous units leads to immediate growth and migratory abnormalities, along with inflammatory changes. The main site of inflammation in AfR is believed to be the pilosebaceous unit. Thus, we considered that AfR was more likely to be exacerbated in individuals with well‐developed sebaceous glands and a perifollicular epidermis. To evaluate without skin measurement instruments, we examined the association between the degree of pore enlargement, oiliness, and redness in facial photographs before EGFR inhibitor administration, and the highest FAfR grade observed in each patient during the corticosteroid treatment period was used. Enlarged skin pores are primarily caused by high sebum excretion, increased hair follicle volume, and decreased elasticity around the pores. Additionally, “stalagmite” structures, epidermal elongations hanging down into the dermis like stalactites, and dermal papillae rising into the epidermis like stalagmites, were observed in enlarged pores [9, 10].

The median time from the initiation of EGFR inhibitor therapy and preemptive treatment to the onset of FAfR and the initiation of corticosteroid therapy was 15.0 days (mean: 18.6 days). Although the precise timing of the highest‐grade FAfR in individual patients was not evaluated, the proportion of patients with FAfR grade 2 or higher was 11.3%, 26.1%, 32.6%, 35.0%, and 35.0% in the ranking‐up group, and 9.8%, 15.7%, 34.0%, 39.5%, and 53.8% in the ranking‐down group at the start of topical corticosteroid therapy and at weeks 2, 4, 6, and 8, respectively [6]. In the FAEISS study [6], which included participants shared with the present study, FAfR severity was centrally assessed by three board‐certified dermatologists using standardized clinical photographs, whereas in the present study, assessment was performed by the attending clinicians.

The present study showed an association between the enlarged pore score and AfR grade; however, no consistent trend was observed between the oiliness score and skin color/redness score. This may be due to the fact that, unlike instrumental measurements, this study employed a rough, naked eye evaluation method, and that the skin surface was no longer oily because the skin was washed before the photographs were taken. Because the pilosebaceous unit is the main site of inflammation in AfR owing to EGFR inhibitors, it is not surprising that the rash is observed in individuals with enlarged pores, in which there is a perifollicular papillomatous epidermis and hypertrophic sebaceous glands.

There was a significant association between skin type with enlarged pores and overall response. However, we were unable to identify confounding factors such as gene mutations in the current study, and it is unclear why patients with skin types with enlarged pores are more likely to respond well to EGFR inhibitors. Further studies are needed to confirm this hypothesis. Additionally, there was no significant association between the worst grade of FAfR and overall response, possibly due to the limited number of patients and short period of therapy in the current study.

In conclusion, this study suggests that the severity of AfR caused by anti‐EGFR antibodies varies with facial skin type, and that facial skin with a high number of enlarged pores is a possible marker for predicting AfR risk due to anti‐EGFR antibody therapy and better therapeutic effects for RAS wild‐type metastatic colorectal cancer.

## 5 Limitations

This study had some limitations. The grading of FAfR was performed by attending physicians, some of whom were non‐dermatologists. Additionally, the facial skin type was assessed by three dermatologists using photographs taken in a standardized manner, which may not have allowed for detailed observation. The overall response to EGFR inhibitors was evaluated at the end of treatment with topical steroid therapy for FAfR, 8 weeks after the second enrollment, or at discontinuation of the protocol therapy in the FAEISS study. The dose intensity of chemotherapy according to skin type has not yet been analyzed.

## Ethics Statement

This study was conducted in accordance with the Declaration of Helsinki and the Japanese Ethical Guidelines for Medical and Health Research Involving Human Subjects. The study protocol was approved by the Ethics Review Board of each participating institution.

## Consent

Each enrolled patient provided written informed consent.

## Conflicts of Interest

The authors declare no conflicts of interest.

## Data Availability

All data generated or analyzed during this study are included in this published article.

## References

[1] D. J. Jonker , C. J. O'Callaghan , C. S. Karapetis , et al., “Cetuximab for the Treatment of Colorectal Cancer,” New England Journal of Medicine 357, no. 20 (2007): 2040–2048. 18003960 10.1056/NEJMoa071834

[2] M. E. Lacouture , E. P. Mitchell , B. Piperdi , et al., “Skin Toxicity Evaluation Protocol With Panitumumab (STEPP), a Phase II, Open‐Label, Randomized Trial Evaluating the Impact of a Pre‐Emptive Skin Treatment Regimen on Skin Toxicities and Quality of Life in Patients With Metastatic Colorectal Cancer,” Journal of Clinical Oncology 28, no. 8 (2010): 1351–1357. 20142600 10.1200/JCO.2008.21.7828

[3] Y. Kobayashi , Y. Komatsu , S. Yuki , et al., “Randomized Controlled Trial on the Skin Toxicity of Panitumumab in Japanese Patients With Metastatic Colorectal Cancer: HGCSG1001 Study; J‐STEPP,” Future Oncology 11, no. 4 (2015): 617–627. 25686117 10.2217/fon.14.251

[4] K. Nishino , Y. Fujiwara , Y. Ohe , et al., “Results of the Non‐Small Cell Lung Cancer Part of a Phase III, Open‐Label, Randomized Trial Evaluating Topical Corticosteroid Therapy for Facial Acneiform Dermatitis Induced by EGFR Inhibitors: Stepwise Rank Down From Potent Corticosteroid (FAEISS Study, NCCH‐1512),” Supportive Care in Cancer 29 (2020): 2327–2334. 32918131 10.1007/s00520-020-05765-7 PMC7981297

[5] K. Kikuchi , K. Nozawa , N. Yamazaki , et al., “Instrumental Evaluation Sensitively Detects Subclinical Skin Changes by the Epidermal Growth Factor Receptor Inhibitors and Risk Factors for Severe Acneiform Eruption,” Journal of Dermatology 46, no. 1 (2019): 18–25. 30402978 10.1111/1346-8138.14691

[6] K. Kikuchi , N. Yamazaki , K. Nozawa , et al., “Topical Corticosteroid Therapy for Facial Acneiform Eruption due to EGFR Inhibitors in Metastatic Colorectal Cancer Patients: A Randomized Controlled Trial Comparing Starting With a Very Strong or a Weak Topical Corticosteroid (FAEISS Study, NCCH1512, Colorectal Part),” Supportive Care in Cancer 30, no. 5 (2022): 4497–4504. 35113224 10.1007/s00520-022-06874-1

[7] M. M. Oken , R. H. Creech , D. C. Tormey , et al., “Toxicity and Response Criteria of the Eastern Cooperative Oncology Group,” American Journal of Clinical Oncology 5, no. 6 (1982): 649–655. 7165009

[8] A. Scope , J. A. Lieb , S. W. Dusza , et al., “A Prospective Randomized Trial of Topical Pimecrolimus for Cetuximab‐Associated Acnelike Eruption,” Journal of the American Academy of Dermatology 61, no. 4 (2009): 614–620. 19646778 10.1016/j.jaad.2009.03.046

[9] S. J. Lee , J. Seok , S. Y. Jeong , K. Y. Park , K. Li , and S. J. Seo , “Facial Pores: Definition, Causes, and Treatment Options,” Dermatologic Surgery 42, no. 3 (2016): 277–285. 26918966 10.1097/DSS.0000000000000657

[10] Y. Sugiyama‐Nakagiri , K. Sugata , M. Iwamura , A. Ohuchi , and T. Kitahara , “Age‐Related Changes in the Epidermal Architecture Around Facial Pores,” Journal of Dermatological Science 50, no. 2 (2008): 151–154. 18249096 10.1016/j.jdermsci.2007.11.014

